# Expression characteristics of circular RNA in human traumatic brain injury

**DOI:** 10.3389/fneur.2022.1086553

**Published:** 2023-01-11

**Authors:** Zhenxing Li, Yixing Lin, Lei Mao, Li Zhang

**Affiliations:** Department of Neurosurgery, Jinling Hospital, School of Medicine, Nanjing University, Nanjing, Jiangsu, China

**Keywords:** human traumatic brain injury, circular RNA, biological processes, KEGG pathway, ceRNA

## Abstract

Traumatic brain injury (TBI) causes high rates of worldwide mortality and morbidity due to the complex secondary injury cascade. Recently, circular ribonucleic acids (circRNAs) have attracted significant attention in a variety of diseases. However, their expression characteristics in human TBI are still unclear. In this study, we examined brain injury tissues from six severe TBI patients in Jinling Hospital. The TBI tissues and adjacent brain contusion tissues were used to analyze differential expression signatures of circRNAs through full-length transcriptome sequencing, Gene Ontology (GO) analysis, Kyoto Encyclopedia of Genes and Genomes (KEGG) pathway analysis, and ceRNA network construction. Our results found that there were 126 differently expressed circRNAs in TBI. Among them, 64 circRNAs were up-regulated and 62 circRNAs were down-regulated. Moreover, GO and KEGG analyses revealed that the aberrantly expressed circRNAs participated in many pathophysiological processes of TBI, especially regarding microglial cell activation, protein transport, protein processing and inflammation. Furthermore, the ceRNA (circRNA-miRNA-mRNA) network predicted that there existed strong relationship among circRNA, miRNA and mRNA. Taken together, our results indicated for the first time that the expression profiles of circRNAs were different after human TBI. In addition, we found the signaling pathways that were related to circRNAs and predicted a ceRNA network, which provided new insight of circRNAs in human TBI.

## 1. Introduction

Traumatic brain injury (TBI) is a leading cause of mortality and disability across all age groups, with estimates of more than 50 million people experiencing TBI each year, imposing a tremendous burden on families and society (1). After the initial insult, the primary injury occurs immediately and can lead to cerebral contusion (2). Although primary injury is the main factor determining the prognosis of TBI patients, the biochemical cascade reactions (secondary brain injury), such as oxidative stress, apoptosis and inflammation, will aggravate the brain tissue damage (3). To date, no effective treatments are available to promote functional recovery after TBI. Therefore, novel therapeutics are significant needed to provide new targets for the treatment of TBI.

Circular ribonucleic acids (circRNAs) are non-coding RNAs derived from back-splicing, which are characterized by a single-stranded, covalently closed-loop structure with no 5' end caps or 3' poly (A) tails (4). CircRNAs can locate in the exon, intron or exon-intron boundary. They are abundantly expressed in eukaryotic cells and demonstrate tissue-specific expression patterns (5). CircRNAs were initially thought to be by-products of transcription with no functions (6). However, increasing evidence have indicated that circRNAs possessed a variety of properties and functions such as regulating gene expression and acting as microRNA (miRNA) sponges, protein decoys and protein translators, making circRNAs as the hotspot of non-coding RNAs research field recently (7, 8). The role of circRNAs in TBI has been studied earlier in animal models. For example, Zhao et al. identified 231 significantly and differentially expressed circRNAs in exosomes from the brain extracellular space after TBI. In further studies they found that these differentially expressed circRNAs might be related to the growth and repair of neurons, the development of the nervous system and the transmission of nerve signals. Moreover, the most highly correlated pathways of these circRNAs were involved primarily with glutamatergic synapse and the cyclic guanosine monophosphate-protein kinase G signaling pathway (9). In another study conducted by Xie et al., they observed that a total of 192 circRNAs were differentially expressed in the rat hippocampus following TBI. Furthermore, Gene ontology (GO) and Kyoto Encyclopedia of Genes and Genomes (KEGG) pathway analysis indicated that many messenger RNAs (mRNAs) transcribed from the host genes of altered circRNAs were implicated in brain damage and neural regeneration. These data demonstrated altered circRNA expression pattern in the rat hippocampus after TBI, which may play important roles in post-TBI physiological and pathological processes (10). The role of circRNAs in TBI has also been confirmed in clinical researches. In the blood of humans with TBI, Zheng et al. detected 3,035 differentially expressed circRNAs in the severe TBI group, 2,362 in the moderate group and 433 in the mild group. Moreover, they constructed a ceRNA network and found that the has_circ_0020269 (circHtra1) was significantly up-regulated after brain insults and was correlated with the severity of injury. In addition, circHtra1 could inhibit cell proliferation and promoted apoptosis, and its knockdown reversed these effects (11).

To date, the expression profiles of circRNAs in human TBI brain tissues has not been fully explained. Therefore, we identified the expression profiles of circRNAs from human TBI in our study.

## 2. Materials and methods

### 2.1. Patient samples

The TBI tissues were extracted from the center of contusion area, and the adjacent brain contusion tissues were taken from the brain tissues 1.5 cm away from the contusion according to our previous study (12). The specimens were immediately frozen in liquid nitrogen and stored at −80°C.

The study protocol was approved by Institutional Review Board of Jinling Hospital (approval number: 2021DZGZR-YBB-082). This study was performed in accordance with the Declaration of Helsinki. A written informed consent to participate and consent for publication were obtained from each patient' surrogates. The clinical data of 6 patients with severe TBI was shown in Table 1. The definition of severe TBI (13), inclusive criteria and exclusion criteria (Brain Trauma Foundation, American Association of Neurological Surgeons and Congress of Neurological Surgeons, 2007) were shown in Table 2.

**Table 1:** Clinical data of six patients with severe TBI.

**Age**	**Sex**	**Lesions**	**GCS (EVM)**
50	Female	Right frontal lobe	7 (E1V2M4)
53	Male	Left frontal lobe	7 (E1V2M4)
59	Male	Left frontotemporal lobe	8 (E2V2M4)
45	Male	Right frontal lobe	7 (E1V2M4)
48	Female	Right frontotemporal lobe	6 (E1V1M4)
57	Male	Right temporal lobe	8 (E2V2M4)

**Table 2:** The definition of severe TBI, inclusive criteria and exclusion criteria in our study.

**The definition of severe TBI**
Large area of brain contusion and laceration, large area of skull fracture, brain stem injury or intracranial hematoma
Coma time > 6 h, Glasgow Coma Score (GCS) was 3–8 points
Obvious nervous system dysfunction and vital signs change
Inclusive criteria
The age of patients was 30–60 years old
GCS was 6–8 points
The cause of TBI was traffic accident
The injury type was brain contusion
The injury site was frontal lobe, temporal lobe or frontotemporal lobe
The injury side was unilateral hemisphere (left or right)
Diagnostic criteria of severe TBI and indications of craniotomy were according to the guidelines
Exclusion criteria
The age of patients < 30 years old or > 60 years old The injury type was laceration, hematoma or skull fracture
The injury site was parietal lobe, cerebellum, or brain stem
The injury side was bilateral hemisphere
Hydrocephalus, cerebral hernia, shock or unstable vital signs
Acute brain swelling during operation
Serious basic diseases (such as heart disease, cancer and thrombocytopenia)
The patient's family members refused the operation

### 2.2. RNA extraction and qRT-PCR

Total RNA was extracted from the brain contusion tissues and adjacent brain contusion tissues using TRIzol regent (Invitrogen, Carlsbad, CA, USA) according to manual instruction. Total RNA was reverse-transcribed using PrimeScript^TM^ RT reagent kit with gDNA Eraser.

qRT-PCR was performed using SYBR^®^ Premix Ex Taq^TM^ II and a 7500 Real-Time PCR System (Applied Biosystems, Foster City, CA, USA) (14). Relative quantification of gene expression was calculated using the formula: 2^−Δ*ΔCt*^, ΔΔCt = (Ct_targetgene_ – Ct_β−*actin*_) injury – (Ct_targetgene_ – Ct_β−*actin*_) injury surrounding. Three independent experiments were performed for each condition.

### 2.3. CircRNA analysis and differential circRNA screening

The Epicentre Ribo-Zero Kit was used to eliminate all ribosomal RNAs from the total RNA (Epicentre). RNase R (Epicentre) digestion was conducted to eliminate all linear RNAs. Finally, pure circRNAs were obtained. After enrichment, the obtained circRNAs were treated with NEBNext^®^ Ultra™ Directional RNA Library preparation kit (Illumina). Finally, the obtained libraries were subjected to paired-end sequencing on an Illumina HiSeq2500 platform (Illumina).

Two algorithms, CIRI2 and CIRCexplorer2 were used to detect circRNAs. The reads were mapped into the human reference genome GRCh37/hg19 (http://genome.ucsc.edu/) by BWA-MEM or Tophat, respectively. CIRI2 detected paired chiastic clipping signals according to the mapping of reads. Systematic filtering in combination with local alignment with BWA-MEM was conducted to eliminate potential false-positive data. CIRCexplorer2 uses Top Hat and Top Hat-Fusion alignment output to detect circRNAs.

If a circRNA was detected by both methods, it was considered to be an identified circRNA. Back-spliced junction reads identified in CIRI2 were integrated and scaled to reads per million (mapped reads, BWA-MEM mapping) to quantify each circRNA. The fold difference of each circRNA was calculated to statistically compare the expression profiles between the TBI tissues and the adjacent brain contusion tissues using Student's *t* test. The differentially expressed circRNAs were defined as *P* < 0.05.

### 2.4. Gene Ontology (GO) and Kyoto Encyclopedia of Genes and Genomes (KEGG) pathway analysis of circRNA

The GO and KEGG pathway analysis were conducted according to previous studies (15). GO enrichment analysis (http://www.geneontology.org) was conducted to explore the potential biological functions of differentially expressed circRNAs. KEGG pathway analysis (http://www.genome.jp/kegg/) was conducted to reveal the signaling pathways of identified circRNAs at the molecular level.

### 2.5. Bioinformatic analysis

The specific binding of circRNAs to miRNAs was predicted by miRanda (http://www.microrna.org/) and PITA (https://genie.weizmann.ac.il/pubs/mir07/mir07_exe.html). The miRNAs-targeted mRNAs were identified by miRanda (http://www.microrna.org/) and Targetscan (http://www.targetscan.org/).

### 2.6. ceRNA (circRNA-miRNA-mRNA) network analysis

According to the predicted targeting relationship among circRNA, miRNA, and mRNA, and the correlation expressed in the samples, the negative correlation between circRNA and miRNA and the negative correlation between mRNA and miRNA are taken to construct the endogenous competitive binding ceRNA (circRNA-miRNA-mRNA) network. Three conditions must exist for the ceRNA network to occur (16). Firstly, the relative concentration of ceRNAs and their miRNAs is clearly important. Secondly, the effectiveness of a ceRNA depends on the number of miRNAs that it can “sponge”. Thirdly, not all of the miRNA response elements on ceRNAs are equal. Therefore, we accept only the ceRNA-pair relations that passed filtering measures (*p* < 0.05). A graph of the circRNA-miRNA-mRNA network was made using Cytoscape software (version 3.5.1) to visualize these relationships.

### 2.7. Statistical analysis

All statistical analysis was performed with SPSS 19.0 (SPSS Inc., Chicago, IL). Data were presented as mean ± SEM. Differences between the TBI group and control group were analyzed using *t*-test. A value of *p* < 0.05 was considered statistically significant.

### 2.8. Database and accession numbers

The circRNA microarray data in our study were deposited at the NCBI GenBank under the Accession No. BankIt2634779: OP794505-OP79462.

## 3. Results

### 3.1. Different express of circRNAs in human TBI tissues and adjacent brain contusion tissues

**Figure 1:**
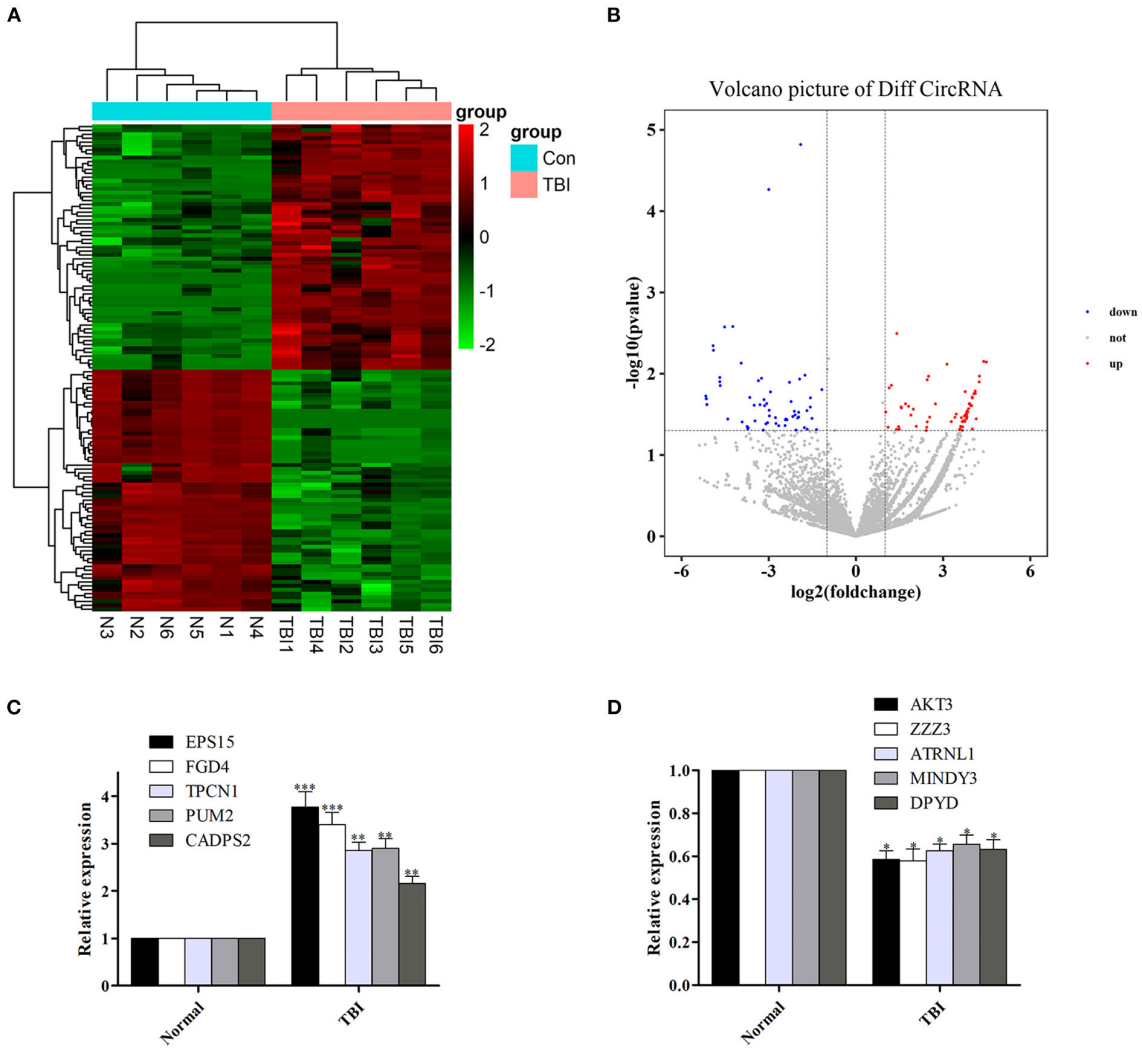
Differentially expressed circRNAs in human TBI tissues, and verify the reliability of sequencing results by qRT-PCR. **(A, B)** Differentially expressed circRNAs in human TBI tissues. **(C)** The expression of the top five up-regulated circRNAs was increased in human TBI tissues. **(D)** The expression of the top five down-regulated circRNAs was decreased in human TBI tissues. *n* = 6 per group. Data are presented as mean ± SEM; ^*^P < 0.05, ^**^P < 0.01, ^***^P < 0.001 vs. adjacent brain contusion group.

Among the 126 differentially expressed circRNAs (*P* < 0.05, Figures 1A, B), 64 were up-regulated and 62 were down-regulated. Among them, circRNA epidermal growth factor receptor pathway substrate 15 (EPS15), FYVE, RhoGEF, and PH domain containing four (FGD4), two-pore channel 1 (TPCN1), PUMILIO 2 (PUM2) and calcium-dependent activator protein for secretion 2 (CADPS2) were top five up-regulated circRNAs, while circRNA serine/threonine protein kinase 3 (AKT3), zinc finger ZZ-type containing 3 (ZZZ3), attractin-like 1 (ATRNL1), motif interacting with Ub-containing novel DUB family 3 (MINDY3) and dihydropyrimidine dehydrogenase (DPYD) were top five down-regulated circRNAs (Table 3).

**Table 3:** Top five up- or down-regulated circRNAs after TBI.

**CircRNA name**	**Gene name**	**Fold change**	***P***
**Top five up-regulated circRNAs**			
chr1:51403418-51406108:-	EPS15	22.42612267	0.007182981
chr12:32598496-32611283:+	FGD4	21.06912332	0.007052801
chr12:113267842-113269845:+	TPCN1	19.02559373	0.010696916
chr2:20307977-20327378:-	PUM2	18.91599313	0.012601556
chr7:122407539-122474517:-	CADPS2	17.62208144	0.035876446
**Top five down-regulated circRNAs**			
chr1:243545509-243695716:-	AKT3	0.444203385	0.015669372
chr1:77631849-77641655:-	ZZZ3	0.390298347	0.048738494
chr10:115120184-115549536:+	ATRNL1	0.350637618	0.035417975
chr10:15833629-15847943:-	MINDY3	0.338159383	0.019803475
chr1:97679094-97721671:-	DPYD	0.337043609	0.025664314

### 3.2. Verify the reliability of sequencing results by qRT-PCR

In order to verify the reliability of sequencing results, we detected the expression levels of the top five up-regulated circRNAs (EPS15, FGD4, TPCN1, PUM2, and CADPS2) and down-regulated circRNAs (AKT3, ZZZ3, ATRNL1, MINDY3, DPYD) in the original samples by qRT-PCR. We found that the expression of the top five up-regulated and down-regulated circRNAs was also increased (Figure 1C) and decreased (Figure 1D) in human TBI tissues, suggesting that the results of qRT-PCR were in agreement with the results of sequencing.

### 3.3. GO and KEGG pathway analyses

**Figure 2:**
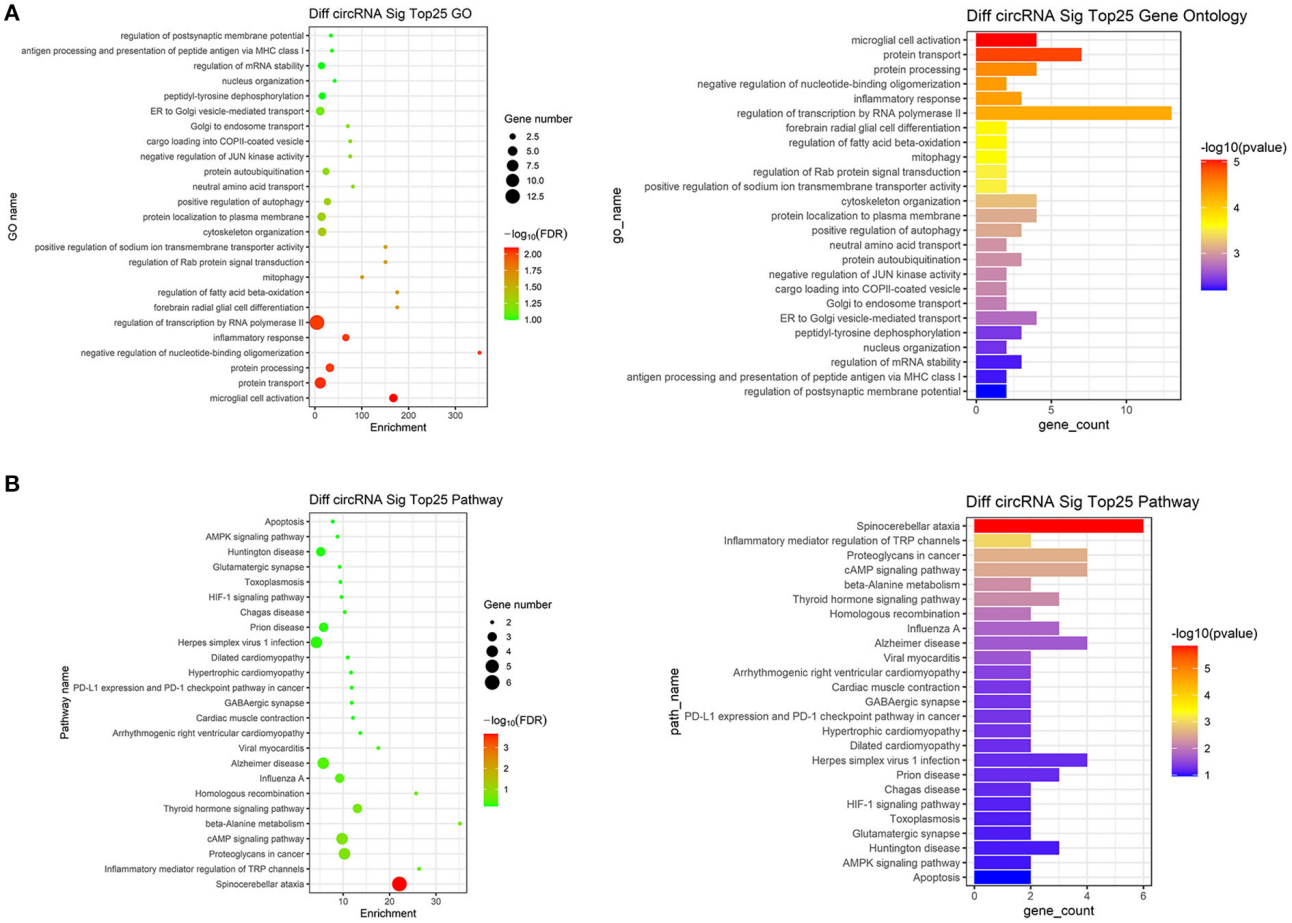
Go analysis and KEGG pathway analysis. **(A)** The top 25 up-regulated and down-regulated biological processes related to differentially expressed circRNAs after TBI. **(B)** The top 25 up-regulated and down-regulated pathways related to differentially expressed circRNAs after TBI.

We performed GO analysis on the differentially expressed circRNAs to reveal the changes of biological process after TBI. We found that the GO were mainly enriched in microglial cell activation, protein transport, protein processing, negative regulation of nucleotide-binding oligomerization and inflammation response (Figure 2A). Subsequently, we analyzed KEGG pathway and found that circRNAs were mainly related to spinocerebellar ataxia, inflammatory mediator regulation of transient receptor potential (TRP) channels, proteoglycans in cancer, cyclic adenosine monophosphate (cAMP) signaling pathway and beta-Alanine metabolism (Figure 2B). The top 25 KEGG pathways that related to target genes after TBI were listed in Table 4.

**Table 4:** The top 25 KEGG pathways related to circRNA after TBI.

**Signaling pathway**	**Count**	***P***
Spinocerebellar ataxia	143	1.41367E-06
Inflammatory mediator regulation of TRP channels	205	0.002619375
Proteoglycans in cancer	216	0.003182128
cAMP signaling pathway	30	0.005994034
beta-Alanine metabolism	121	0.006508165
Thyroid hormone signaling pathway	40	0.010615703
Homologous recombination	41	0.011146303
Influenza A	171	0.017278636
Alzheimer disease	369	0.021980429
Viral myocarditis	60	0.023501708
Arrhythmogenic right ventricular cardiomyopathy	77	0.038045782
Cardiac muscle contraction	87	0.048048654
GABAergic synapse	89	0.050172924
PD-L1 expression and PD-1 checkpoint pathway in cancer	89	0.050172924
Hypertrophic cardiomyopathy	90	0.051250249
Dilated cardiomyopathy	96	0.057924241
Herpes simplex virus 1 infection	498	0.060867285
Prion disease	273	0.061596332
Chagas disease	102	0.064951918
HIF-1 signaling pathway	109	0.07358626
Toxoplasmosis	112	0.077427048
Glutamatergic synapse	114	0.080033574
Huntington disease	306	0.083052103
AMPK signaling pathway	120	0.088070548
Apoptosis	128	0.099282367

### 3.4. miRNA prediction and ceRNA network construction

**Figure 3:**
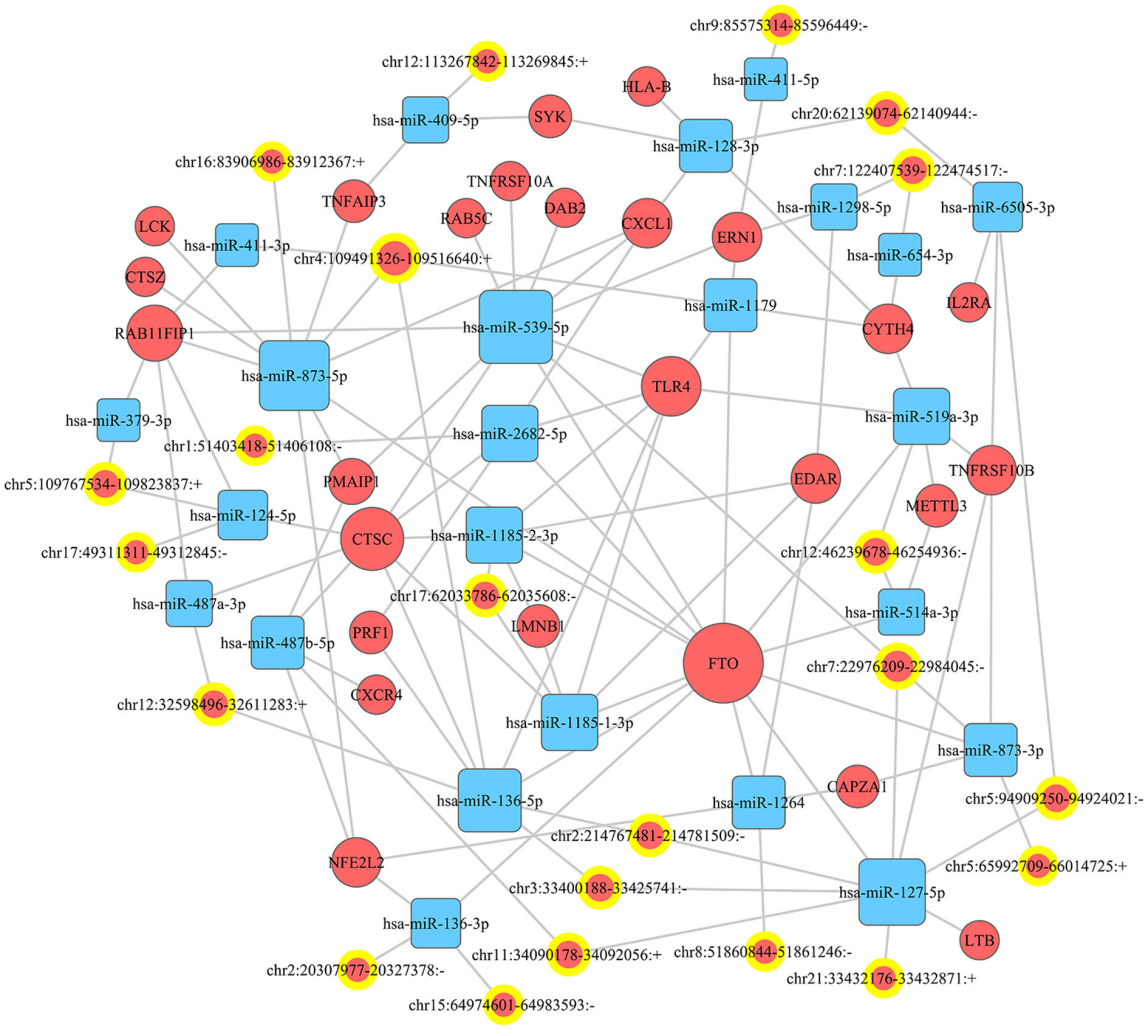
CeRNA network. A ceRNA (circRNA-miRNA-mRNA) network was established to show the relationship among circRNA, miRNA, and mRNA.

miRanda and PITA were used to predict miRNAs targeted by the differentially expressed circRNAs. The miRNAs predicted to be targeted by the top five significantly differentially expressed circRNAs, which were all contained in the two databases, are presented in Table 5. The miRNA-mRNA regulatory relationships were further identified using miRanda and Targetscan. The circRNA-targeted miRNAs and their regulated mRNAs were further selected for ceRNA network construction (Figure 3).

**Table 5:** Predicted miRNAs for the selected significantly differential circRNAs in TBI.

**circRNA name**	**Gene name**	**miRNA**
chr1:51403418-51406108:-	EPS15	hsa-miR-2682-5p
chr12:32598496-32611283:+	FGD4	hsa-miR-487a-3p, hsa-miR-136-5p
chr12:113267842-113269845:+	TPCN1	hsa-miR-409-5p
chr2:20307977-20327378:-	PUM2	hsa-miR-136-3p
chr7:122407539-122474517:-	CADPS2	hsa-miR-654-3p, hsa-miR-1298-5p

## 4. Discussion

In the present study, we found that: (1) there existed differentially expressed circRNAs between human TBI tissues and adjacent brain contusion tissues; (2) GO and KEGG analyses indicated that the aberrantly expressed circRNAs mainly participated in microglial cell activation and inflammation; (3) the circRNAs-targeted miRNAs and miRNAs-targeted mRNAs were predicted; (4) a ceRNA network was established.

It has been shown that circRNAs participated in the pathophysiological process of CNS diseases (5, 17). However, the role of circRNAs in TBI was unknown. Some circRNAs have been proposed which may explain the effects of circRNAs in TBI. In a mouse TBI model, circHtra1 could promote neuronal loss and immune deficiency in TBI (11). Moreover, knockdown of circRNA_009194 improved neurological outcomes in TBI (18). Furthermore, inhibition of circIgfbp2 alleviated mitochondrial dysfunction and oxidative stress-induced synapse dysfunction after TBI (19). In our study, we examined circRNAs expression profiles from six TBI patients. A total of 126 circRNAs (64 up-regulated and 62 down-regulated) were differentially expressed between server TBI tissues and adjacent brain contusion tissues. Furthermore, we found that the five top up-regulated circRNAs were EPS15, FGD4, TPCN1, PUM2 and CADPS2, and the five top down-regulated circRNAs were AKT3, ZZZ3, ATRNL1, MINDY3 and DPYD. Among them, circRNA EPS15 owned the maximized up-regulated fold change. CircRNA EPS15 is derived from the parent gene Eps15. Eps15 is transcribed from ch38. p13, which encodes a component of the epidermal growth factor receptor (EGFR) pathway, exists in the pits of the reticular protein coating, and participates in the receptor-mediated endocytosis of EGF (20). To date, the top five up/down-regulated circRNAs have not been reported in previous studies. We would confirm the role of these circRNAs in TBI in our future researches.

In order to understand these differentially expressed circRNAs, the possible biological functions of their parent gene were analyzed by GO and KEGG analysis. GO analysis showed that the mostly enriched GO were located in the microglial cell activation, protein transport, protein processing, negative regulation of nucleotide-binding oligomerization and inflammation response. Based on KEGG results, the most enriched pathways were spinocerebellar ataxia, inflammatory mediator regulation of TRP channels, proteoglycans in cancer, cAMP signaling pathway and beta-Alanine metabolism. From GO and KEGG analysis, we found that microglial cell activation-induced inflammation response was very critical in TBI. Microglial cells, derived from myelogenous cells, support physiological processes by secreting neurotrophic factors and assisting with the formation and elimination of synapses (21). Moreover, microglial cells provide immune responses in respond to a number of stimuli (22). After TBI, microglia are activated, the activated microglia secrete a large number of neuroimmune inflammatory factors such as interleukin-1β (IL-1β), IL-10 and tumor necrosis factor-α (TNF-α), thus exacerbating the inflammatory response and leading to a series of brain injuries, including cognitive impairment, blood-brain barrier (BBB) disruption and brain edema (23, 24). Recently, Eps15, the parent gene of circRNA EPS15, has also been suggested to regulate microglial cell activation and inflammation. It has been found that in intracerebral hemorrhage (ICH), microglial activation marker CD68 was co-located with EHD2, a member of the Eps15 homology domain (EH domain) family (25). Moreover, the Eps15 homology domain-containing protein 1 (EHD1) could mediate the formation of functional IL-6 receptor complexes through D4-positive raft (26). In addition, knockdown of Eps15 significantly decreased endocytosed particulate matter (PM) and reduced the PM-induced production of IL-6 (27). Moreover, cAMP pathway is another significant signaling cascade in TBI. cAMP signaling cascade is one of the most essential and best-understood cellular pathways which mediate the action of a wide variety of hormones, neurotransmitters, and growth factors (28). Besides, the presence of cAMP is essential for the control and regulation of gene expression, growth, cell differentiation as well as proliferation and apoptosis (29). The versatility of cAMP-related effects depends on the expression of many factors such as adenylyl cyclase isoform, phosphodiesterase and A kinase-anchoring proteins (AKAP) (30).

A great number of evidence have suggested that circRNAs can serve as ceRNAs for miRNAs, namely, as miRNA sponges. A circRNA may contain multiple miRNA binding sites and may have adsorptive and suppressive effects on miRNAs. For example, in acute lymphoblastic leukemia (ALL), circ_0000745 knockdown restrained cell cycle progression, and triggered cell apoptosis and ferroptosis. Circ_0000745 acted as a molecular sponge for miR-494-3p in ALL cells and miR-494-3p silencing partly diminished circ_0000745 knockdown-induced anti-tumor effects (31). In addition, in prostate cancer (PC), circPDHX deficiency attenuated PC cell proliferation, migration and fatty acid metabolites. Moreover, circPDHX could bind to miR-497-5p to act as ceRNA (32). In our study, the potential target genes of circRNAs were examined to investigate the functions of these circRNAs. We firstly predicted miRNA candidates based on MiRanda and PITA, and listed the miRNA candidates for the top 5 differentially expressed circRNAs (EPS15, FGD4, TPCN1, PUM2 and CADPS2). Then, the target-binding mRNAs for miRNA candidates were predicted *via* miRanda and Targetscan. In our study, the ceRNA (circRNA-miRNA-mRNA) network was constructed, which presented a large interaction network for bioinformatic analysis. These results together lay the groundwork for future research into the specific ceRNA network, which is beneficial for investigating the role of circRNAs in regulating the expression of target genes. From the ceRNA network, wo found that fat mass and obesity-associated protein (FTO) was the key mRNA that could be regulated by a lot of miRNAs and circRNAs. FTO is the first discovered demethylase that belongs to the alpha-ketoglutarate-dependent dioxygenase B (ALKB) family. It is an oxygenase dependent on Fe^2+^ and 2-oxoglutarate, which can catalyze the demethylation of nucleotides (33). FTO-mediated demethylation process is gradually completed. M6A is first converted to hm6A, then transformed to fm6A, and finally reduced to original adenylate (34, 35). FTO is enriched in brain, especially in neurons, and plays an important regulatory role in central nervous system (CNS), including brain development and function (36). Dysregulation of FTO may participate in CNS injuries such as TBI. Yu et al. found that the expression of FTO was significantly down-regulated in rat cerebral cortex after TBI. Moreover, functional FTO was necessary to repair TBI-induced neurological damage (37).

There were some limitations in our study. Firstly, we did not confirm the regulatory relationship of circRNA, miRNA and mRNA *via* gain and loss experiment in our study. Secondly, we did not examine the role of circRNA in animal TBI models, which we would further study in our future experiments. Thirdly, we did not validate whether the signaling pathways found in our study by KEGG was involved in animal TBI models. Fourthly, the six severe TBI patients were chosen only in Jinling Hospital of China, and nothing about other countries, so our study might not be representative.

In conclusion, our study indicated that the expression of circRNAs was significantly different in TBI. Moreover, microarray, proteomic and metabolomic analyses of the downstream moleculars of circRNAs may offer new avenues for restoring normal neuronal network and blocking the vital nodes promoting brain damage. We considered that circRNAs can to be attractive therapeutic targets for patients suffering from TBI. Continued discoveries in this field will bring novel insights on circRNAs involved in biological functions and disease progression. Ultimately, circRNAs may hold promise for clinical challenges.

## Data availability statement

The data presented in the study are deposited in the NCBI GenBank repository, accession number BankIt2634779: OP794505-OP79462.

## Ethics statement

The studies involving human participants were reviewed and approved by Jinling Hospital (approval number: 2021DZGZR-YBB-082). The patients/participants provided their written informed consent to participate in this study. Written informed consent was obtained from the individual(s) for the publication of any potentially identifiable images or data included in this article.

## Author contributions

ZL was responsible for the data collection and manuscript writing. YL was responsible for the clinical specimen collection and proofreading. LM was responsible for the literature collection and manuscript review. LZ was responsible for the design of the article. All authors contributed to the article and approved the submitted version.
